# Clinical and computational development of a patient-calibrated ICGFA bowel transection recommender

**DOI:** 10.1007/s00464-024-10827-6

**Published:** 2024-04-18

**Authors:** Jeffrey Dalli, Jonathan P. Epperlein, Niall P. Hardy, Mohammad Faraz Khan, Pol Mac Aonghusa, Ronan A. Cahill

**Affiliations:** 1 UCD Centre for Precision Surgery, School of Medicine, University College Dublin, Catherine McAuley Centre, 21 Nelson St, Dublin 7, D07 KX5K Ireland; 2 Department of Surgery, Mater Misericordiae University Hospital, Dublin, Ireland; 3 IBM Research Europe, Dublin, Ireland

**Keywords:** Near infrared, Indocyanine green, Colorectal cancer

## Abstract

**Introduction:**

Intraoperative indocyanine green fluorescence angiography (ICGFA) aims to reduce colorectal anastomotic complications. However, signal interpretation is inconsistent and confounded by patient physiology and system behaviours. Here, we demonstrate a proof of concept of a novel clinical and computational method for patient calibrated quantitative ICGFA (QICGFA) bowel transection recommendation.

**Methods:**

Patients undergoing elective colorectal resection had colonic ICGFA both immediately after operative commencement prior to any dissection and again, as usual, just before anastomotic construction. Video recordings of both ICGFA acquisitions were blindly quantified post hoc across selected colonic regions of interest (ROIs) using tracking-quantification software and computationally compared with satisfactory perfusion assumed in second time-point ROIs, demonstrating 85% agreement with baseline ICGFA. ROI quantification outputs detailing projected perfusion sufficiency-insufficiency zones were compared to the actual surgeon-selected transection/anastomotic construction site for left/right-sided resections, respectively. Anastomotic outcomes were recorded, and tissue lactate was also measured in the devascularised colonic segment in a subgroup of patients. The novel perfusion zone projections were developed as full-screen recommendations via overlay heatmaps.

**Results:**

No patient suffered intra- or early postoperative anastomotic complications. Following computational development (*n* = 14) the software recommended zone (ROI) contained the expert surgical site of transection in almost all cases (Jaccard similarity index 0.91) of the nine patient validation series. Previously published ICGFA time-series milestone descriptors correlated moderately well, but lactate measurements did not. High resolution augmented reality heatmaps presenting recommendations from all pixels of the bowel ICGFA were generated for all cases.

**Conclusions:**

By benchmarking to the patient’s own baseline perfusion, this novel QICGFA method could allow the deployment of algorithmic personalised NIR bowel transection point recommendation in a way fitting existing clinical workflow.

**Supplementary Information:**

The online version contains supplementary material available at 10.1007/s00464-024-10827-6.

Bowel resection remains important in the management of colorectal diseases, and restoring continuity is preferred whenever possible, especially by patients [1]. As anastomotic breakdown and enteric content leakage results in morbidity, mortality, poor oncological outcomes and increased costs [2, 3] it is important to optimise anastomotic construction by ensuring technically sound, tension free apposition of well perfused intestinal tissue. With the mechanical components addressed by standardised technique, colonic perfusion sufficiency judgement remains the most subjective controllable variable and is now attracting significant clinical focus.

Near infrared cameras (NIR) permit the visualisation of circulating fluorescent dyes, thus enabling intraoperative colonic perfusion assessment via indocyanine green fluorescence angiography (ICGFA). This technique has been mostly demonstrated to cost-effectively diminish complications [4, 5] including in a large scale clinical trial [6] and NIR capability has become common in contemporary surgical camera systems. However, despite efforts to standardise its use [7], technique heterogeneity is common and training remains experiential [8, 9]. In addition, NIR sensing, and presentation varies among systems and optical technical considerations such as target positioning can complicate fluorescence signal interpretation [10–12]. Furthermore, the fluorescence signal is itself dependent on ICG’s pharmacokinetics which in turn vary with body habitus [13], physiology [14] and intra-operative factors, including anaesthesia [15]. Nevertheless, interpretation consistency did not improve when a protocol was applied to standardise these variables relating to the technique, as well patient and camera positioning [16]. Overall, these factors hinder interpretative consistency and limit technique dissemination and uptake, undermining the potential for ICGFA to become the gold standard clinical method for intraoperative perfusion assessment [8, 17, 18].

ICGFA signal quantification (QICGFA) [19] allows enumeration of variations in fluorescence intensity (a surrogate for ICG tissue concentration), which can be translated into curves and may facilitate dynamic fluorescence imagery interpretation. Retrospective correlation of extracted curve features has indicated associations with anastomotic leakage (AL) [20, 21], and explorative machine learning (ML) models have also been trained [22]. However, it has been difficult to fully benchmark these to intraoperative metabolic or postoperative outcomes as surrogates of tissue perfusion. As factors impacting human visual assessment also complicate computational perfusion assessment [10–12], a clinically meaningfully grounded QICGFA method that compensates for such variability is needed.

Here, we develop and present a novel clinical and computational methodology for automated QICGFA transection recommendation based on a personalised ICGFA perfusion signature generated from the patient’s own bowel perfusion at an earlier intraoperative time point. We subsequently compare its outputs to expert user ICGFA interpretation (previously proven as a consistent parameter among experienced ICGFA surgeons) and other previously attempted perfusion surrogates.

## Methodology

**Fig. 1:**
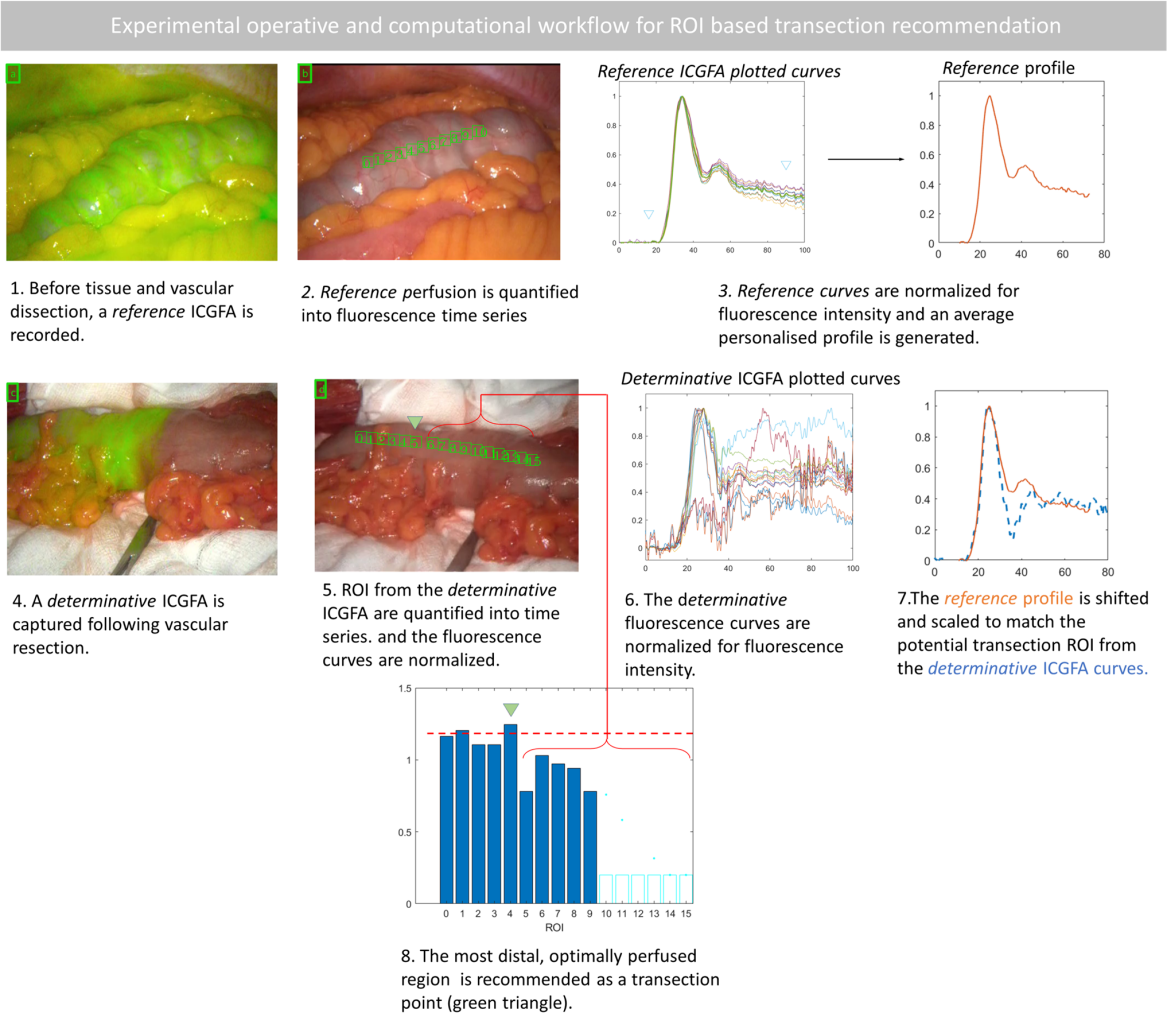
The experimental operative and computational workflow. Curves denote time in seconds versus normalised fluorescence ratio. The photographs show still video frames with overlaid NIR and white light imagery from both (**a** and **b**) the *reference* and (**c** and **d**) *determinative* ICGFAs

Within a trial (institutional review board 1/378/2092 Dublin, Ireland; ClinicalTrials.gov identifier: NCT04220242), consenting adult patients undergoing elective right and left sided colonic resection had a baseline colonic ICGFA performed early in the operation to determine normal intestinal perfusion prior to segmental devascularisation. This was mathematically compared to the more usual perianastomotic ICGFA (see Fig. 1), which in standard practice is the first time ICGFA is performed intraoperatively. The operative team was not blinded to the ICGFA appearances and used the latter ICGFA to confirm their planned transection point, as is routine. While some residual fluorescence may persist in the bowel wall, redosing after five minutes has been demonstrated to permit refluorescence of the target tissue, with such repeat ICGFA being commonly practised after anastomotic construction [23].

ICGFA computational analyses were performed post hoc on ICGFA recordings, blinded to where the intraoperative transection was performed. Computational predictions of regions of sufficient/insufficient perfusion were generated by mathematically comparing the perianastomotic ICGFA time series with the baseline ICGFA. Following a clinical series for software development, such computational projections were validated in a subgroup against expert surgical user judgement (i.e. the site actually selected for transection), tissue lactate [24] and other previously described QICGFA parameters [9, 19, 25, 26].

### Patients and procedures

All patients undergoing elective right and left sided colonic resections during the study timeframe were eligible for inclusion. Procedures were performed by a colorectal specialist team led by a consultant with expertise in ICGFA as previously described [8]. Clinical data including patient demographics and postoperative outcomes were accrued from patient records.

### Operative and ICGFA method

After initial laparoscopy and port placement, and before any intestinal tissue dissection, an immediate colonic ICGFA was acquired as a *reference* ICGFA. To achieve this, a relevant region of bowel was visualised (i.e. a segment of bowel likely near where the ultimate transection would be performed) and following peripheral intravenous injection of 0.1 mg/kg ICG an angiogram was recorded over four minutes (to capture inflow and early outflow [27, 28]) via a commercially available laparoscopic stack that provides synchronous white light and NIR display of the tissue under observation (PINPOINT, Stryker, USA). Subsequent routine colonic mobilisation and operative dissection of the mesentery was carried out as per standard practice, including distal transection either extracorporeally or intracorporeally as per the surgeon’s preference. Extracorporeally, the bowel was delivered via a small, central laparotomy with a wound protector-retractor (Alexis, Applied Medical, USA) and laid on surgical gauze. The mesentery dissection was completed, and the operator determined their planned transection point.

Subsequently, a second *determinative* ICGFA (again 0.1 mg/kg, with dimmed lights if acquired extracorporeally) was performed of either the ileum and colon for right-sided resections or of the proximal colonic segment for left sided resections and recorded.

The angiogram was visually interpreted in real-time by the operating surgeon and a clinical decision made as usual by the surgeon with the video recording including the site of elective transection. The operation progressed to conclusion either with extracorporeal side-to-side or intracorporeal end-to end colorectal stapled anastomosis for proximal and distal resections, respectively.

### Fluorescence quantification

Recorded ICGFA videos (30 frames per second) were analysed after the surgery using software developed by IBM Research Ireland [29, 30]. This software tracks the bowel in the simultaneously presented white light image and quantifies the fluorescence intensity displayed on the synchronous NIR display. A time series is generated from the operative video for user annotated (via a line on the bowel serosa) and subsequently computationally spaced regions of interest (ROI) for both *reference* and *determinative* ICGFA recordings.

### ROI-based recommendation

**Fig. 2:**
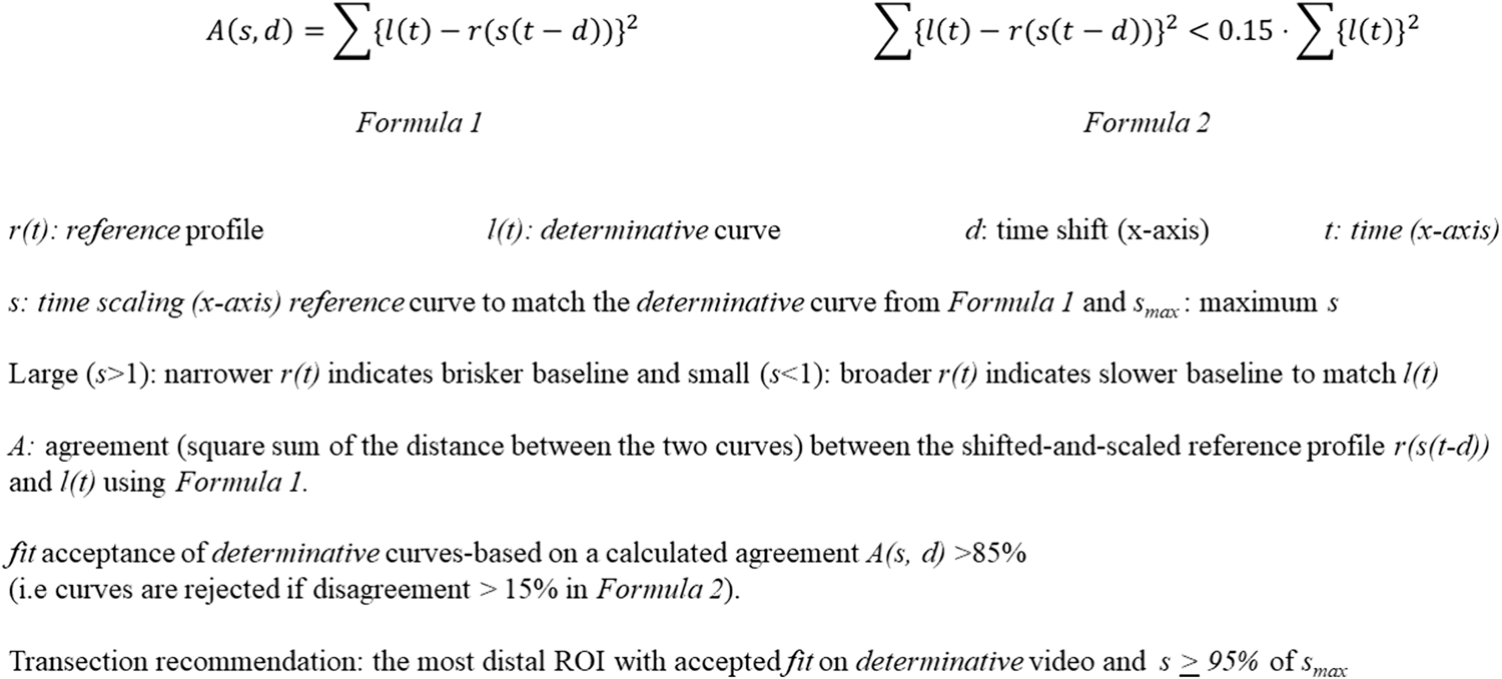
The mathematical formulae (1 and 2), which were used to determine ROI-based transection recommendation

Utilising MATLAB® R2022 (MathWorks®, USA) any background fluorescence was filtered out and curves were normalised and also smoothed via Savitzky-Golay [31] filter. The quantified fluorescence time series from the *reference* video was plotted as a *reference* profile reflecting the patient’s normal colonic circulation under anaesthesia. The shape and chronology of the *reference* curve was adapted to the second ICGFA *determinative* curve by scaling and shifting it along the x-axis to maximise agreement between the shifted-and-scaled reference profile (see Formula 1 Fig. 2). Narrowing of the *reference* curve to match the *determinative* one indicates a brisker baseline perfusion, while slower perfusion in the baseline is indicated by any requirement of the *reference* curve to be broadened to match the *determinative* ICGFA curve (Formula 1). *Determinative* curves were identified as adequate/inadequate based on *fit* with respect to the reference curves. Curves were rejected if calculated agreement was less than 85% of the *determinative* curve (see Formula 2 Fig. 2). From the included curves, perfusion sufficiency was assumed at the briskest (or 95% as fast as the briskest) *determinative* curves. Transection recommendation was recommended at the most distal, sufficiently perfused ROI from the *determinative* video.

### Validation series

For the validation subgroup (including only extracorporeal cases) the prepared exteriorised bowel was marked at 2 cm intervals on the antimesenteric border in its devascularised segment at and distal to the surgeon’s planned transection. The outputs of ROI-based recommendation were compared to three different metrics, namely surgeon judgement, previously reported QICGFA descriptors and lactate measurement.

### Comparison with surgeon’s transection decision

The intraoperative surgical transection was compared to the postoperatively computationally identified regions having adequate curve *fit*. The level of agreement between the two was calculated using the Jaccard similarity index [32].

### Comparison with QICGFA descriptors

Previously reported quantitative metrics [9, 19, 25, 26] were identified from published literature and calculated from the fluorescence time series curves at the pre-determined 2 cm gradations on the *determinative* ICGFA recordings in this study. These metrics included inflow parameters, namely *latency* for pre-inflow period, peak intensity (*Fmax*), the time to achieve this peak from the end of *latency (Tmax)* and *upslope* gradient. Outflow parameters included the overall *downslope,* as well as the intensity and gradient at fifty and one hundred seconds post peak. Both putative and previously described complex computational parameters (respectively *centre of mass* [25] and *time ratio* TR: *T½*:*Tmax* where *T½* denotes the time required to achieve half *Fmax *[20]) were also investigated.

### Metabolic testing

The serosa was incised using a blade down to muscularis propria to induce bleeding at the marked 2 cm intervals (see supplementary material). This blood was sampled using a handheld lactate analyser (The Edge, Apex Bio, Taiwan). To control for time-related variations in sampling, incision and measurement was carried out both proximal to distal and distal to proximal alternatively. Absolute (mg/dl) and relative ratios of lactic acid concentrations from the portion of bowel planned for excision were correlated to outputs of Formula 1 for each ROI.

Correlations were performed, via Spearman’s correlation (rho < 0.1 “negligible”, 0.1–0.39 “weak”, 0.4–0.69 “moderate”, 0.7–0.89 “strong”, > 0.89 “very strong” [33], significance *p* < 0.05) in SPSS Version 27 (IBS, USA) and these were correlated to curve *fit* rejection, *s,* and difference in curve areas.

### Development of high resolution (per pixel) quantification and recommendation

The MATLAB® code was advanced to permit video stabilisation and more detailed quantification, enabling time series quantification from every pixel (rather than from ROIs). Thereafter, initial heatmap generation was carried out, demonstrating *Fmax* and *Tmax* calculated from every pixel and presented with colour gradients representing the values overlaid on the white light displayed image (first frame of the video). For transection recommendation based on Formula 1, the antimesenteric region of the colon was annotated, and the calculation was applied to the extracted time series from every pixel within the annotated region. Curves with a good *fit* via Formula 2 (see Fig. 2) were plotted as a histogram in relation to *s* with transection recommendation based on the curve distribution of *s *(see Supplementary Material).

To allow for the possibility that portions of the onscreen bowel may have better perfusion profiles than the *reference* curve, the 75th percentile was selected as the threshold. Recommendation was then applied to indicate sufficient (with green highlighting curves with 25% of the 75th percentile), insufficient (with red highlighting curves in the lowest quartile or those rejected via Formula 2, see Fig. 2) or borderline (with orange displaying curves in between 25 to 75% of the 75th percentile, see supplementary material).

## Results

23 patients were recruited into the study (see Table 1) with data from 14 used to develop the computational methodology and nine used to validate it. The development series included 12 with extracorporeal determinative ICGFA and 2 with intracorporeal ICGFA. There were no ICG related, other intraoperative, or early (within 30 days) postoperative complications (although one patient undergoing anterior resection following neoadjuvant therapy had an incidentally detected, subclinical leak seen on surveillance CT scan and proven on gastrograffin enema one year post surgery).

**Table 1:** shows demographics for the patients in the development series, for all patients (inclusive of validation subgroup) and only for the validation subgroup

Patient and analysis data			
	All	Development series	Validation subgroup
*N*	23	14	9
Mean age	63.91	65.6	61.44
Male: female	16:07	12:02	04:05
Cancer: benign	15:08	09:05	06:03
T Stage	T4(3) T3(5) T2(5) T1(2)	T4(2) T3(4) T2(2) T1(1)	T4(1) T3(1) T2(3) T1(1)
Left: right	15:08	08:06	07:02
Extracorporeal: intracorporeal	21:02	12:02	9:00
Bowel continuity restored	22 of 23	13 of 14	9 of 9
Large: small bowel analysis	23:07	14:05	09:02

**Fig. 3:**
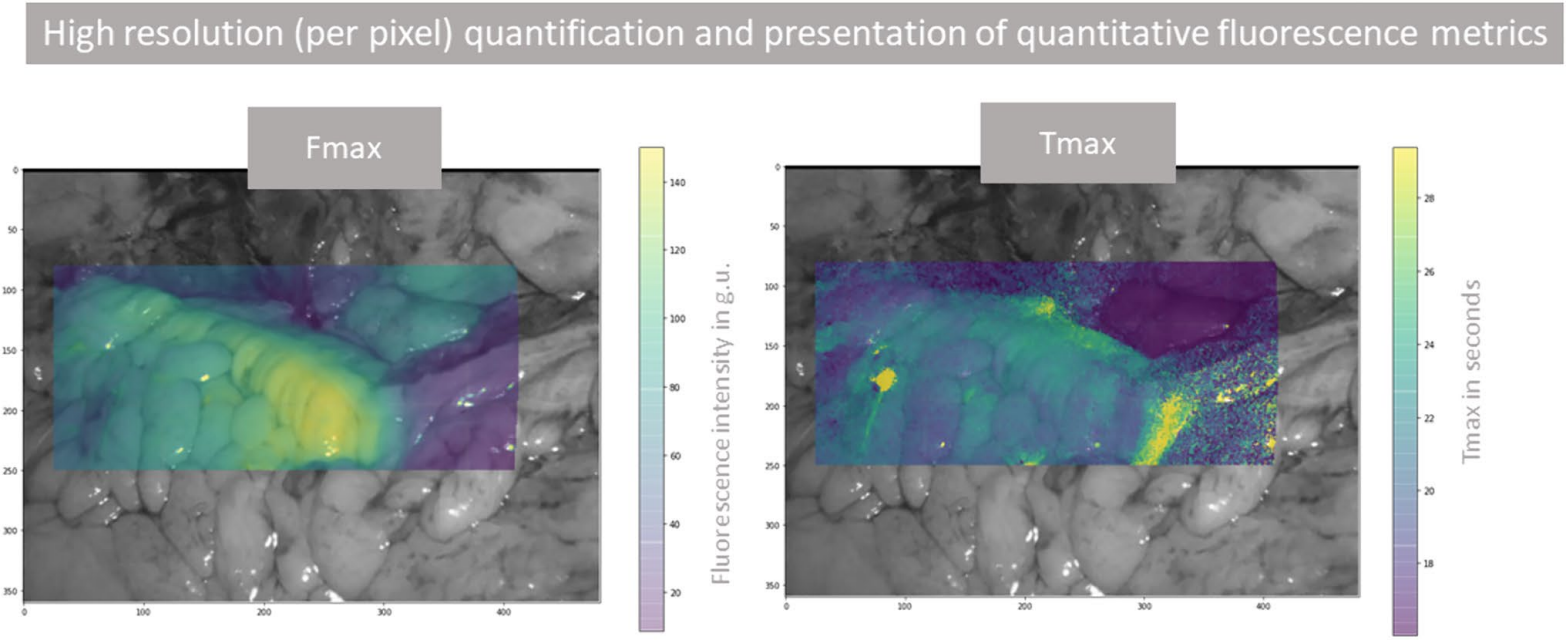
An illustrative high resolution (per pixel) quantification and presentation of quantitative fluorescence metrics (*Fmax* and *Tmax*) heatmap on the *determinative* ICGFA assessment of the descending colon for a left hemicolectomy for cancer. The legend on the left shows the colour scale for each plot (ranges vary by panel) with yellow being the highest value (brightest or slowest) and purple being the least (darkest or slowest). The axes denote co-ordinates on the screen. These metrics do not take into consideration the *reference* angiogram

**Fig. 4:**
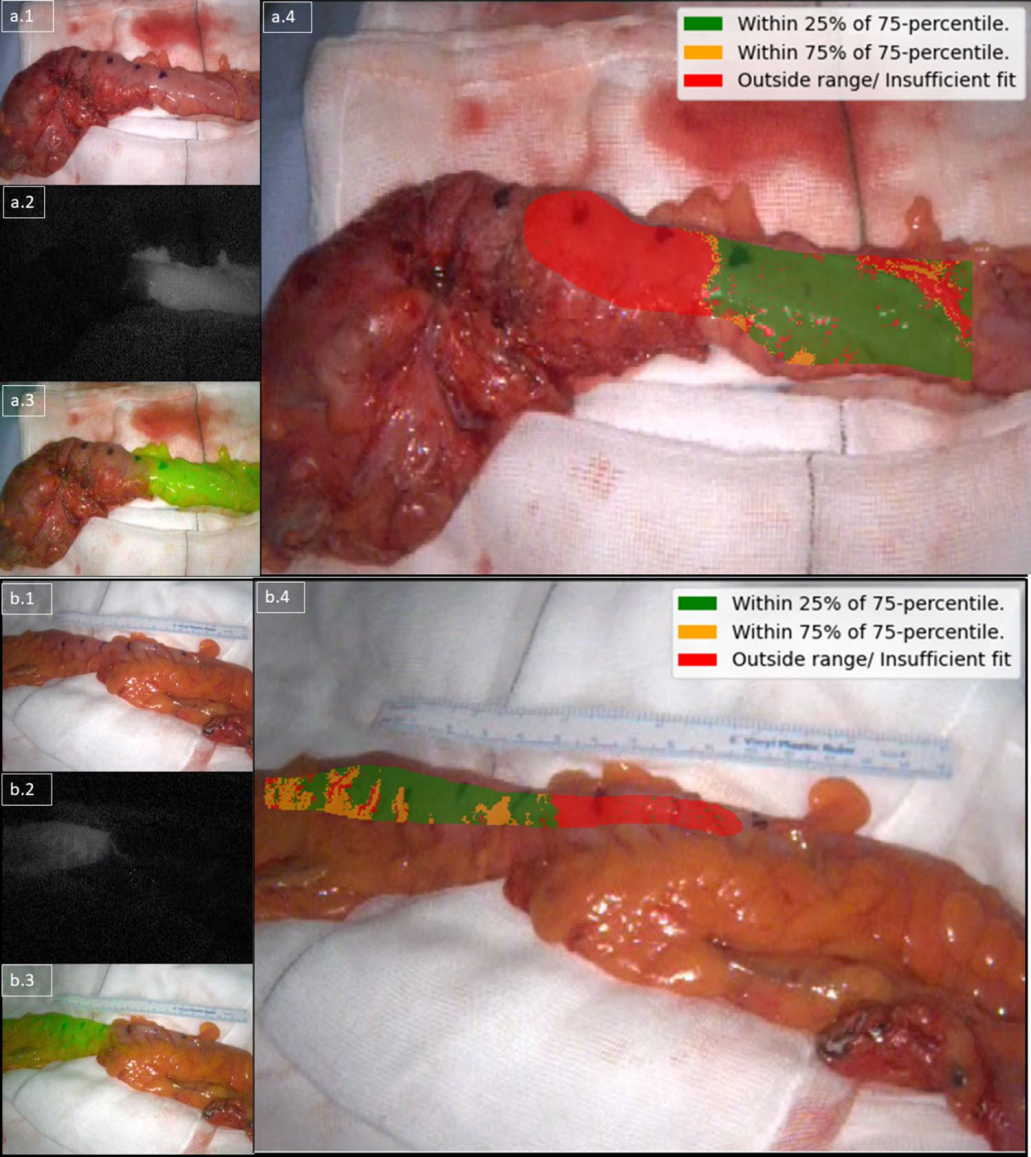
A composite image illustrating an augmented reality per pixel transection recommendation using Formula 1 for a sigmoid resection for diverticular disease on top (**a**, proximal on the right) and for T1N1 adenocarcinoma below (**b**, proximal on the left). The panel on the top left (**a.1** and **b.1**) shows the white light view, the one in the middle left (**a.2** and **b.2**) shows the raw NIR feed, and the ones on the bottom left (**a.3** and **b.3**) show an overlay view (composed of the NIR feed overlaid on the white light feed). These three images are unchanged from the display of the camera system. The main large panels on the right (**a.4** and **b.5**) show the computed recommendation augmented on the white light view. Computational recommendation (legend top right of **a.4** and **b.4**) with green indicating sufficient, orange borderline and red insufficient perfusion (see supplementary material)

Recording, tracking, and quantification was achieved for all videos with postoperative analysis generating a total of 3.1 million data instances. *Reference* profiles were synthesised from 246 ROIs and *determinative* profiles from 266 ROIs, permitting automatic selection of the most distal well perfused ROI in all patients including the small (*n* = 6) and large bowel (*n* = 22, see Table 1, Fig. 1 and supplementary material) for both intra and extracorporeal assessments except for a single instance. For this case, a right hemicolectomy, the software did not identify a suitable transection location on the ascending colon but did so on the ileum. Automated quantification on augmented reality heatmaps were generated for previously described QICGFA metrics (see Fig. 3), and also for the proposed transection recommendation methodology (see Fig. 4).

For most cases within the validation series, the expert selected transection site matched the zones (ROI) identified by the software as having adequate *curve fit* (*n* = 10 of 11 judgments, including both transections for right hemicolectomies on *n* = 9 patients). This resulted in a Jaccard similarity coefficient of 0.91. For two of these cases, the algorithm also recommended a more distal safe transection zone.

Regarding metabolic validation, mean lactate concentrations were greater in the ascending versus descending colon samples (1.67 ± 0.5 vs 1.24 ± 0.8 mg/dl *p* = 0.04) but neither concentrations nor ratios significantly differed with either direction of sampling or small versus large bowel. When comparing the metabolic validation data with the data computed from Formula 1 (see Fig. 2), there were no relevant correlations (see Table 2). Curve fit acceptance from Formula 1 (see Fig. 2) demonstrated moderate correlation with previously described upslope and as *downslope* metrics (slopes at fifty and a hundred seconds 0.62–0.64 *p* < 0.001). When comparing the metabolic data with previously reported QICGFA metrics, lactate concentration demonstrated *moderate* negative correlations with *downslope* (− 0.64) and lactate ratio with *Fmax* (− 0.56 both *p* < 0.001).

**Table 2:** displays bivariate Spearman's rank correlation coefficients (* denotes *p* < 0.05, two-tailed) for the results from metabolic validation in comparison to both the metrics from the proposed Formula 1 (see Fig. 2) as well as previously described QICGFA

Spearman’s rank correlation coefficients																			
	Metabolic		New formula				Conventional QICGFA												
	Lactate (mg/dl)	Lactate ratio	Scale factor	Diff. in curve area	Fit peak	Fit accepted	Upslope	T1/2	*Fmax*	*Tmax*	Dslope	T50	F50	D50	T100	F100	D100	TR	C.M
Lactate concentration (mg/dl)	1.00	0.61*	− 0.10	0.29	0.16	− 0.48*	− 0.27	− 0.22	− 0.50*	− 0.21	− 0.64*	0.46*	− 0.43*	− 0.56*	0.46*	− 0.48*	− 0.51*	0.19	0.32*
Lactate ratio	0.61*	1.00	0.22	0.32*	0.04	− 0.49*	− 0.31*	− 0.20	− 0.56*	− 0.22	− 0.50*	0.33*	− 0.48*	− 0.50*	0.33*	− 0.51*	− 0.49*	0.26	0.15
Scale factor	− 0.10	0.22	1.00	0.16	− 0.58*	0.04	0.16	− 0.08	− 0.09	− 0.06	0.02	− 0.34*	− 0.15	0.00	− 0.34*	− 0.13	− 0.01	− 0.13	− 0.21
Diff. in curve area	0.29	0.32*	0.16	1.00	0.44*	− 0.74*	− 0.48*	− 0.45*	− 0.62*	− 0.45*	− 0.36*	0.30*	− 0.63*	− 0.55*	0.30*	− 0.53*	− 0.64*	0.42*	− 0.04
fit peak	0.16	0.04	− 0.58*	0.44*	1.00	− 0.48*	− 0.42*	− 0.16	− 0.21	− 0.18	− 0.15	0.61*	− 0.16	− 0.31*	0.61*	− 0.12	− 0.33*	0.40*	0.28
Fit accepted	− 0.48*	− 0.49*	0.04	− 0.74*	− 0.48*	1.00	0.64*	0.43*	0.54*	0.43*	0.52*	− 0.44*	0.46*	0.62*	− 0.44*	0.43*	0.64*	− 0.51*	− 0.19
Upslope	− 0.27	− 0.31*	0.16	− 0.48*	− 0.42*	0.64*	1.00	0.70*	0.56*	0.70*	0.41*	− 0.32*	0.46*	0.53*	− 0.32*	0.52*	0.52*	− 0.92*	− 0.01
T1/2	− 0.22	− 0.20	− 0.08	− 0.45*	− 0.16	0.43*	0.70*	1.00	0.62*	0.99*	0.37*	− 0.01	0.66*	0.36*	− 0.01	0.68*	0.37*	− 0.72*	0.16
*Fmax*	− 0.50*	− 0.56*	− 0.09	− 0.62*	− 0.21	0.54*	0.56*	0.62*	1.00	0.64*	0.51*	− 0.37*	0.94*	0.82*	− 0.37*	0.93*	0.86*	− 0.60*	0.21
*Tmax*	− 0.21	− 0.22	− 0.06	− 0.45*	− 0.18	0.43*	0.70*	0.99*	0.64*	1.00	0.37*	− 0.02	0.68*	0.37*	− 0.02	0.70*	0.38*	− 0.74*	0.17
Downslope	− 0.64*	− 0.50*	0.02	− 0.36*	− 0.15	0.52*	0.41*	0.37*	0.51*	0.37*	1.00	− 0.14	0.42*	0.52*	− 0.14	0.45*	0.49*	− 0.29	− 0.30
T50	0.46*	0.33*	− 0.34*	0.30*	0.61*	− 0.44*	− 0.32*	− 0.01	− 0.37*	− 0.02	− 0.14	1.00	− 0.27	− 0.47*	10.00*	− 0.27	− 0.51*	0.30	0.50*
F50	− 0.43*	− 0.48*	− 0.15	− 0.63*	− 0.16	0.46*	0.46*	0.66*	0.94*	0.68*	0.42*	− 0.27	1.00	0.61*	− 0.27	0.97*	0.68*	− 0.50*	0.27
D50	− 0.56*	− 0.50*	0.00	− 0.55*	− 0.31*	0.62*	0.53*	0.36*	0.82*	0.37*	0.52*	− 0.47*	0.61*	1.00	− 0.47*	0.62*	0.97*	− 0.53*	0.05
T100	0.46*	0.33*	− 0.34*	0.30*	0.61*	− 0.44*	− 0.32*	− 0.01	− 0.37*	− 0.02	− 0.14	10.00*	− 0.27	− 0.47*	1.00	− 0.27	− 0.51*	0.30	0.50*
F100	− 0.48*	− 0.51*	− 0.13	− 0.53*	− 0.12	0.43*	0.52*	0.68*	0.93*	0.70*	0.45*	− 0.27	0.97*	0.62*	− 0.27	1.00	0.65*	− 0.55*	0.26
D100	− 0.51*	− 0.49*	− 0.01	− 0.64*	− 0.33*	0.64*	0.52*	0.37*	0.86*	0.38*	0.49*	− 0.51*	0.68*	0.97*	− 0.51*	0.65*	1.00	− 0.52*	0.06
TR	0.19	0.26	− 0.13	0.42*	0.40*	− 0.51*	− 0.92*	− 0.72*	− 0.60*	− 0.74*	− 0.29	0.30	− 0.50*	− 0.53*	0.30	− 0.55*	− 0.52*	1.00	− 0.11
C.M	0.32*	0.15	− 0.21	− 0.04	0.28	− 0.19	− 0.01	0.16	0.21	0.17	− 0.30	0.50*	0.27	0.05	0.50*	0.26	0.06	− 0.11	1.00

*Diff* difference, *T* time, *F* fluorescence, *D* downslope, 50 and 100 denote time after *Tmax*. *TR* is a previously described ratio of *T1/2* and *Tmax*, *C.M.* centre of mass

## Discussion

Although clinical trials supporting ICGFA are accumulating, issues relating to interpretation variation and equipment behaviour may undermine clinical uptake [8, 11]. Indeed, the lack of standardised objective interpretation may have contributed to some studies failing to show significant benefit [34, 35]. QICGFA signal and computational interrogation could help address interuser variability and learning curve considerations, but its clinical application requires that this data itself has meaning as an objective, relevant indicator of perfusion sufficiency or indeed insufficiency. Such benchmarking needs meaningful intraoperative or postoperative correlation. While previous studies have focussed on postoperative outcomes, this is difficult and imperfect as AL is uncommon (requiring large cohorts [36]) and may occur for other reasons. Therefore, it would seem better to benchmark against a relevant intraoperative measurement.

Previously, we [23] and others have used another portion of the bowel as a simultaneous on-screen reference [37]. However, there are physiological issues with comparing small bowel perfusion with that of the large bowel, since its ICGFA perfusion differs from the colon. While this work also demonstrates assessment of both large and small bowel, recommendations were only based on comparisons of the same bowel type. Indeed, simultaneous on-screen comparisons save time compared to the proposed method, however, the NIR signal in the periphery of the screen is weaker and this may complicate visual and quantitative comparisons [11, 12] of bowel in different potions of the screen. It may also be impractical to visualise two distinct colonic segments in this way.

Although methods of profiling tissue oxygen or ischaemic metabolites are proposed, these are difficult to acquire, and their clinical meaning is uncertain. Tissue lactate sampling requires the induction of bleeding via serosal incision (as needling alone was found to be insufficient). In addition, this sampling can only be carried out extracorporeally and distal to the already planned transection line. Furthermore, this technique has only been previously described in an animal model, allowing much greater ischaemia than experienced clinically [38]), while mitochondrial respiration assessment necessitates full thickness biopsies. Non-invasive tissue oxygen saturation hyperspectral oximetry measurement [39] has potential but is still of uncertain clinical relevance and there is currently no CE-marked device for use in Europe [40, 41].

Here, rather than trying to link such extrapolated surrogates, we compare the *perianastomotic* ICGFA to an earlier time point in the same operation (and so person) when the bowel perfusion had not been surgically compromised. To accomplish this, mathematical methods are used to scale and compare the two curves and a threshold of similarity (set to 95%) to the earlier curve taken to indicate sufficiency of the latter curves. The generated heatmaps provide a usable surgical interpretation of these calculations.

For almost all cases, this method reflected actual expert surgical judgement (the clinical gold standard and one proven reliable and consistent [8, 16] with regard to ICGFA interpretation). Although these recommendations are not clinically validated in this retrospective in silico research, the software also recommended a more distal safe transection in two patients. Such options would be helpful when colonic length needs to be preserved.

Besides the encouraging expert-based comparisons, the formula also showed moderate correlation with previously described curve parameters (which potentially reflects the uncertainty in value of these other methods). There was no correlation with lactate, however, perhaps reflective of the variability inherent in such measurement.

Indeed, the only discordance occurred where the software offered no recommendation. For this case, this *determinative* ICGFA appeared dim, and the quantified signal computed a rough (noisy) curve, potentially compromising the comparison. It has been, in fact, demonstrated that this camera and 30-degree scope lens setup have a narrow field of view, diminishing NIR performance when held at the distance required to extracorporeally assess sizable portions of bowel [11]. Mathematical signal normalisation and a focus on the timing of the curve rather than the absolute brightness sought to address any differences between the fluorescence environments of the intra and extracorporeal assessments. However, the enclosed peritoneal cavity presents specific optical parameters and outside the abdomen other factors such as ambient light [42] (e.g. from anaesthetic machines) may also impact the fluorescence signal. There could also be distortion of the bowel dimensions when the bowel is assessed outside the abdominal cavity, complicating comparisons. Further development could include rejection of suboptimal ICGFA, or use of systems featuring dual laparoscopic and dedicated open setups (assuming consistent fluorescence performance across both setups, which is not always the case [11]).

While this work involved a clinical series of patients undergoing a step change in how ICG is presently used, repeat dosing is within licence and common anyway in colorectal surgery although usually the perianastomotic dose represents the first acquisition with subsequent doses coming after step level change or anastomotic construction rather than performing the index ICGFA earlier in the operation. While some background fluorescence can be retained between the doses, the time required for oncological resection exceeds the half-life of ICG of 3–5 min [27] and the inter-dose period is considerably longer in our method versus that in standard practice (bowel mobilisation can take an hour or more while further anastomotic preparation after bowel transection typically only takes a few minutes). The method described here so calibrates the recommendation to the patient’s own specific bowel perfusion profile, allowing an objective comparison of a similarly selected and positioned segment of colon at two operative time points. This seeks to circumvent inter patient variations in physiology impacting ICGFA. However, this does not factor in intra-operative variations (e.g. due to anaesthetic drugs) which would require real time splanchnic perfusion monitoring during both ICGFA recordings. However, commercially available pulse spectrophotometry devices (e.g. LiMON, Getinge, Sweden) only output summarising values and not comparative curves.

This study is a retrospective developmental series and at present only offers a proof of concept, as in our small dataset no patient suffered clinically important anastomotic ischaemia. Thus, appropriate validation would require a prospectively powered non-interventional study to assess outcomes on a cohort large enough to feature complications. Furthermore, the baseline assumption of this work is that ICGFA indeed reflects clinically important tissue perfusion accurately. The limited numbers also precluded a comparison of how the tool performed on different bowel types. Moreover, while the operator was blinded from the mathematical recommendations as this analysis took place postoperatively, these predictions have only been validated against a single expert’s judgement.

Further development could permit intraoperative recommendation by installing the software on a computer in theatre or potentially even on the laparoscopic system to provide intraoperative ROI and high-resolution recommendation from the ICGFA. While ROI recommendations are straightforward, the generated heatmaps require user interpretation assessment prior to clinical deployment. Clinical use would also benefit from a user interface that can be operated while maintaining sterility via gestures or voice.

Although based on mathematics, our tool’s functionality does meet certain criteria defining artificial intelligence [43] and thus future development and deployment should follow DECIDE-AI [44]. Besides these technical considerations, clinical pathways also need to be developed to provide investigators or clinicians with options to undertake when the tool reports that there is no suitable transection. This may include further resection or proceeding with anastomotic reconstruction. An appreciation of the tool’s performance parameters would also support the clinician to correlate computational recommendations with their clinical judgement.

## Conclusion

While the novel quantitative metrics presented here require further validation and clinical correlation, the proposed clinical and computational workflow has been shown to allow feasible personalised algorithmic NIR bowel transection point recommendation.

### Supplementary Information

Below is the link to the electronic supplementary material.

Supplementary file1 (TIF 19227 kb)

Supplementary file2 (TIF 1753 kb)

Supplementary file3 (TIF 1985 kb)

Supplementary file4 (DOCX 13 kb)
