# Supplementary figures and images for: Clinical and computational development of a patient-calibrated ICGFA bowel transection recommender
Source: Surg Endosc. 2024 Apr 18;38(6):3212–22. doi: 10.1007/s00464-024-10827-6 (PMC11133155; doi:10.1007/s00464-024-10827-6)

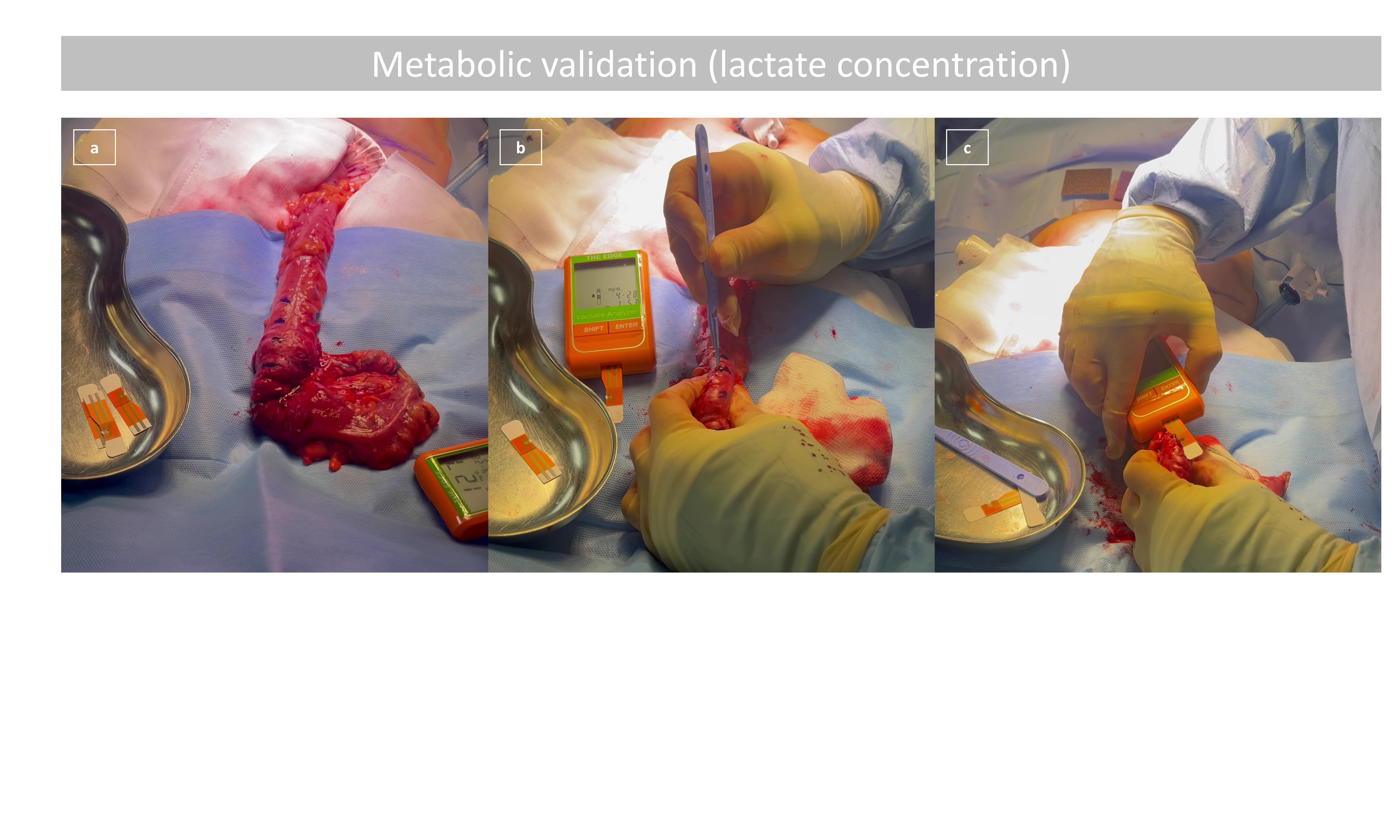

Supplement: Supplementary file 1 — Supplementary file1 (TIF 19227 kb) [file 464_2024_10827_MOESM1_ESM.tif]

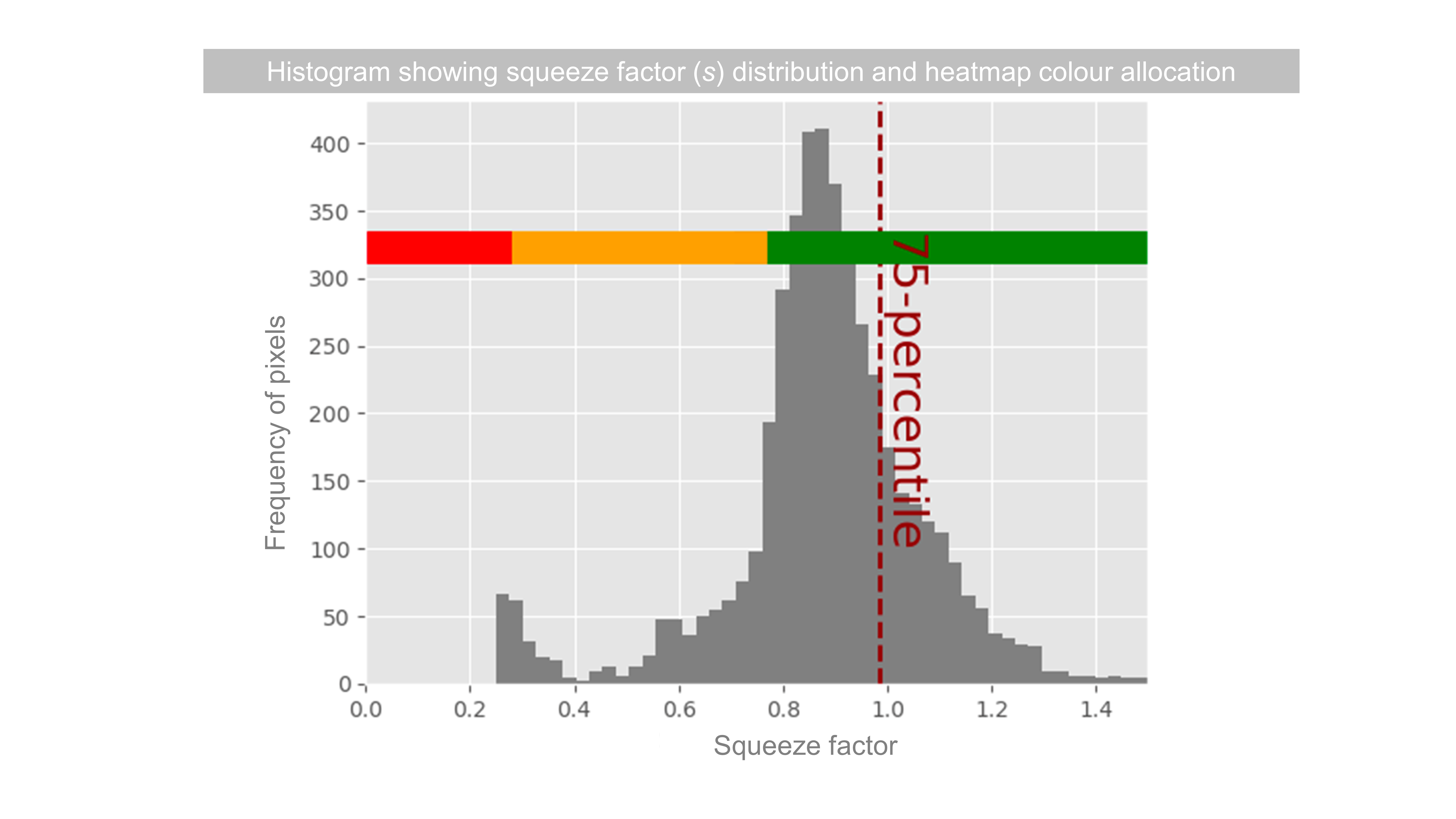

Supplement: Supplementary file 2 — Supplementary file2 (TIF 1753 kb) [file 464_2024_10827_MOESM2_ESM.tif]

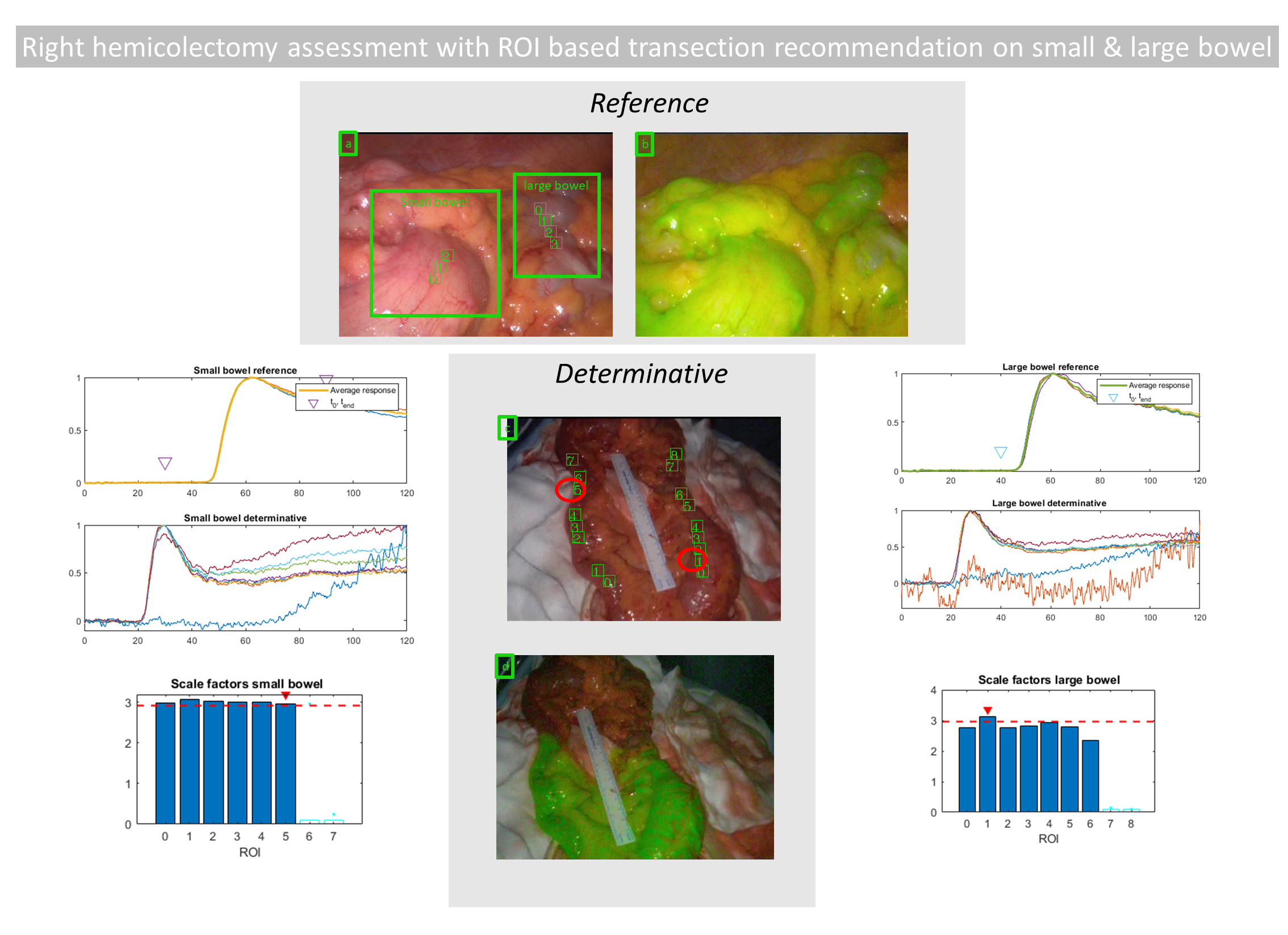

Supplement: Supplementary file 3 — Supplementary file3 (TIF 1985 kb) [file 464_2024_10827_MOESM3_ESM.tif]
