# Supplementary material for: Clinical and computational development of a patient-calibrated ICGFA bowel transection recommender
Source: Surg Endosc. 2024 Apr 18;38(6):3212–22. doi: 10.1007/s00464-024-10827-6 (PMC11133155; doi:10.1007/s00464-024-10827-6)
Supplement: Supplementary file 4 — Supplementary file4 (DOCX 13 kb) [file 464_2024_10827_MOESM4_ESM.docx]

# Supplementary figures

Supplementary figure 1 demonstrates the process of extracorporeal lactate sampling at 2 cm intervals, at and distal to the point at which the mesentery was prepared prior to *determinative* ICGFA for a sigmoid resection for diverticular disease (panel a). The bowel was incised using a blade (b) to expose the muscularis propria and induce bleeding. Tissue lactate concentration was measured at these intervals using a handheld lactate analyser (c).

Supplementary figure 2 illustrates squeeze factor *s* distribution as calculated by Formula 1. Fitted curves were subdivided in relation to the 75th percentile (marked here with a vertical red dotted line). Pixels with curves within 25% of the 75th percentile (and better) were labelled as green, those out with that group but within 75% of the 75th percentile orange. The remaining pixels with the lowest squeeze factors (lower quartile) and curves which did not meet fit criteria by Formula 2 were labelled red.

Supplementary figure 3: For a right hemicolectomy this compound figure shows *reference* assessment of the caecum(large) and ileum (small bowel) with ROI annotation on the white light image (a, and a still image from the corresponding ICGFA: b) for the creation of a *reference* profile. Following the *determinative* extracorporeal ICGFA assessment, annotated ROI (c, white light image) are converted into time series from the angiogram (representative still ICGFA image: d). The *reference* profile is scaled and shifted to match the curves and an optimal ROI transection recommendation is made on the bar graphs showing scale factor versus ROI for small (left) and large (right) bowel (inverted red triangle). These recommendations are illustrated via red circles on the white light *determinative* image (c). *T* denotes time at the start (0) and at the end of the detected curve.
